# INVITED COMMENTARY on Andersen S, et al. Developmental Windows of Environmental Vulnerability for Inflammatory Bowel Disease

**DOI:** 10.1016/j.jpedcp.2024.200104

**Published:** 2024-03-16

**Authors:** David A. Simon, Richard Kellermayer

**Affiliations:** 1 Division of Pediatric Gastroenterology, Texas Children’s Hospital Baylor College of Medicine, Houston, TX; 2 USDA/ARS Children’s Nutrition Research Center, Houston, TX

Inflammatory bowel diseases (IBDs: Crohn’s disease [CD] and ulcerative colitis [UC]) are disorders of relative wealth and education in the background of westernization.^1^ The incidence of IBD has increased greatly with economic advancement around the world.^2^ The disease group is just one example for the increase of autoimmune disorders in countries adopting a westernized lifestyle,^3^ implicating shared genetic^4^ but more importantly common environmental^3^^,^^5^ origins. The environmental (including dietary, pharmaceutical, etc) shifts of the past 60-70 years in economically advanced countries were tremendous and multifactorial, many of which have been proposed to partake in the etiology of IBD^6^ and autoimmunity.^5^ One of these factors, which has been implicated by several studies, is early postnatal exposure to antibiotics leading to increased risk for subsequent IBD, most convincingly that of CD.^7^^,^^8^

In this volume of *The Journal of Pediatrics: Clinical Practice*, Andersen and colleagues examined the relationship between fetal and early postnatal antibiotic exposure and subsequent IBD, including all children of Norway born 2004-2012 until the study end of December 31, 2020.^9^ The large-scale epidemiologic work included 797 cases of pediatric inflammatory bowel disease (PIBD) among 536 819 children and found that early postnatal antibiotic exposure significantly, but relatively subtly (aOR 1.33), was associated with PIBD. The association was driven by the cases of CD and was not significant for UC or IBD unclassified. These findings are in line with previous epidemiologic work.^7^^,^^8^ Among the potential confounders examined, maternal smoking had the largest effect, which has been observed to be a risk factor for IBD (only CD)^10^ and might modify disease behavior as well.^11^ Although socioeconomic status showed no significant influence on the outcomes, Norway’s esteemed social justice system poses challenges for studying this environmental factor compared with other economically advanced countries, where a significant association between PIBD and household income has been established.^1^ Overall, Andersen et al^9^ provide further evidence for the microbial developmental origins of disease,^12^ highlighting age-related windows of environmental vulnerability,^13^ and indicating the disease-specific nature of individual environmental insults in PIBD pathogenesis. Animal models support such epidemiologic observations by demonstrating that antibiotic exposure during a critical window of mammalian development has lasting metabolic consequences^14^ and can increase colitis susceptibility later in life.^15^

A major challenge for maternal fetal and pediatric medicine is to develop strategies of disease prevention during such vulnerable developmental windows against harmful iatrogenic (such as antibiotics) and other environmental insults to promote future health and to counteract the increasing incidence of autoimmunity around the world. Antibiotic stewardship (https://www.cdc.gov/antibiotic-use/core-elements/outpatient.html) is of major importance but may only provide modest and selective (such as against CD but not UC, for example) protection against autoimmune disorders.

We have recently introduced the concept of 3 (prenatal, perinatal/early-postnatal, and pediatric/young adult) developmental windows of environmental vulnerability (ie, the “triple environmental hit”) in the pathogenesis of IBD^13^ and other autoimmune disorders.^5^ Interactive epigenetic-immune-microbiome changes, as the result of their stochastic and environmentally sensitive nature,^6^^,^^16^ appear to be cooperatively involved in the autoimmune phenotype shift of humanity in the 20th/21st century.^5^ Importantly, recognizing environmentally vulnerable developmental widows in disease pathogenesis creates potential opportunities for prevention. We have examined these developmental windows in murine models of colitis and raised potential modes for prevention, as examples:

1. Prenatal window (Figure, point 1): the environmentally sensitive mammalian epigenome postfertilization is a prime candidate in the developmental origins of human disease,^16^ including IBD.^17^, ^18^, ^19^ We have shown that prenatal methyl donor supplementation, which modulates DNA methylation (a key mechanism in epigenetic regulation), increases colitis susceptibility in young adult mice.^20^ Furthermore, the prenatal dietary intervention induced the postnatal nurturing of a colitis-prone microbiome.^21^ Based on our studies, we proposed that the select and timely use of prenatal vitamins (such as folic acid during the first trimester) as opposed to their constant consumption throughout pregnancy may be preferable.^22^ Further maternal fetal research is critical for the development of prenatally preventative interventions against autoimmune disease.^23^

**Figure:**
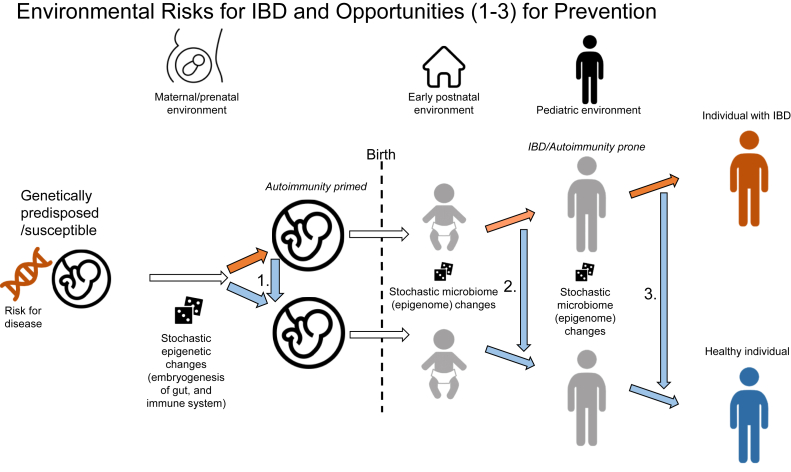
Visual representation of vulnerable developmental windows (prenatal/perinatal [point 1], early postnatal [point 2], and pediatric [point 3]) implicated in the pathogenesis of IBD. These windows may also provide opportunities for disease prevention in individuals at increased risk by shifting disease-associated environmentally induced prenatal epigenetic priming (*arrow 1*), early postnatal microbiome and epigenome changes (*arrow 2*), and pediatric gut microbiome-immune modifications (*arrow 3*) toward health. See text for details and examples.

2. Perinatal and early postnatal window (Figure, point 2): the epigenetic evolution of the gut mucosa continues into pediatric development,^24^ although at lower velocity than during the prenatal period.^25^ This postnatal epigenetic maturation is closely associated with the establishment^26^ and evolution of the gut microbiome.^25^ It is in this dynamic, partly stochastic, cooperative host-commensal-microbiome development^6^ where postnatal environmental insults such as antibiotics can leave long-lasting marks, thereby leading to an IBD/autoimmune disease-prone individual.^5^^,^^12^ Considering the stochastic nature of a colitis-prone microbiome development, we have recently identified a potentially anti-inflammatory 3-strain *Lactobacillus reuteri* probiotic combination.^27^ This combination, if delivered through maternal supplementation predelivery and during lactation (ie, in the perinatal and infant developmental window), could protect female offspring from colitis in young adulthood. Such research can provide support and nidus toward developing carefully designed postnatal microbiome targeted interventions as prevention against disease later in life.

3. Pediatric window (Figure, point 3): although the evolution of the human gut microbiome is most dynamic before 3 years of age, a slower, essentially lifelong shift occurs in it,^12^^,^^28^ likely in close interaction with our immune system.^29^ Therefore, there is a window of opportunity for pediatric environmental insults to push a genetically susceptible, prenatally primed, postnatally prone individual toward developing IBD or other autoimmune diseases during adolescence or young adulthood through possible microbiome-epigenome interactions^25^ upon a critical trigger. Our animal model and epidemiologic work has furthered the potential protective role of dietary fiber (cellulose),^30^ monotonous diets,^31^^,^^32^ large households,^33^ and avoidance of chronic stress^10^^,^^34^ during pediatric development against IBD. Such studies can inform us of possible diet, microbiome, and lifestyle-centered preventative interventions against IBD. IBD epidemiology-based predictions for disease prevention, however, need careful preclinical investigation. An example is our work on transient exposure to a high omega-6 fat–supplemented diet in mice during pediatric development, which was based on epidemiologic findings indicating polyunsaturated fatty acid intake to significantly contribute to the developmental origin of IBD. As opposed to expected, transient obesity secondary to omega-6 fat supplementation during childhood protected young adult mice against colitis. Interestingly, we demonstrated that this protection could be transmitted by the microbiome.^35^ This finding furthers the importance of combating pediatric obesity and implicates that such interventions may protect against autoimmune disorders as well. Furthermore, our environmental-developmental-origin/environmental-window approach toward omega-6 fat diet during childhood pointed to the CXCL13-CXCR5 pathway as a potential novel therapeutic target in IBD,^35^ with subsequent observations supporting our discovery.^36^^,^^37^

We congratulate Andersen et al for their important work,^9^ which highlights the need for not only antibiotic stewardship but also for increased research focus on prenatal and postnatal developmental windows of opportunity for prevention against autoimmune disorders. Such studies may not only direct disease prevention but also identify novel therapeutic avenues.

## CRediT authorship contribution statement

**David A. Simon:** Writing – original draft. **Richard Kellermayer:** Conceptualization, Supervision, Writing – review & editing.

## Declaration of Competing Interest

R.K. was supported by the Gutsy Kids Fund, generously maintained by Brock Wagner, the Klaasmeyers, the Frugonis, and other donor families. The authors declare no conflict of interest.
